# A study of the impact of DIP payment reform on coronary heart disease hospitalization costs and equity

**DOI:** 10.3389/fpubh.2025.1567838

**Published:** 2025-06-09

**Authors:** Yingying Tao, Keyi Shen, Yating Chen, Chengcheng Li, Dan Wu, Xuehui Meng

**Affiliations:** School of Humanities and Management, Zhejiang Chinese Medical University, Hangzhou, China

**Keywords:** DIP payment reform, interrupted time series analysis, types of health insurance, healthcare cost control, healthcare equity

## Abstract

**Background:**

To control the growth of healthcare costs, the Chinese government introduced a diagnosis-intervention package (DIP)-based health insurance payment reform. This study evaluated the impact of the DIP policy on hospitalization costs, Length of Hospital Stay, and Out-of-Pocket Ratio for patients with coronary heart disease (CHD).

**Methods:**

Hospitalization claims data from 2020 to 2023 in City S, central China, were selected and analyzed using interrupted time series (ITS), covering 264 hospitals with January 2022 as the intervention point.

**Results:**

After the implementation of DIP, hospitalization costs decreased from 8.81 to 8.57 for employee health insurance (UEBMI) (*p* < 0.001) and from 8.18 to 7.97 for resident health insurance (URRBMI) (*p* < 0.001), with even greater decreases for primary and secondary hospitals. The number of days of hospitalization decreased, from 8.82 to 7.78 (*p* < 0.001) for UEBMI and from 8.24 to 7.46 (*p* < 0.001) for URRBMI, with the largest decrease in primary hospitals. As for out-of-pocket ratio, the URRBMI increased from 20.71 to 25.2% (*p* < 0.001), and the UEBMI decreased from 28.67 to 23.57% (*p* < 0.001).

**Conclusion:**

The DIP policy was effective in controlling hospitalization costs and days, especially in primary and secondary hospitals. However, the out-of-pocket ratio of URRBMI increased and UEBMI decreased, suggesting differential impact of the policy. It is recommended that policy makers pay attention to differences in health insurance types and hospital grades to optimize the fairness and effectiveness of the policy.

## 1 Introduction

Coronary heart disease (CHD) is a prevalent cardiovascular disease globally, characterized by high morbidity and mortality rates, which pose significant threats to human health and life safety (1). In China, the increasing prevalence of CHD is attributed to population aging and widespread unhealthy lifestyles, resulting in a yearly rise in the number of affected individuals (2). Hospitalization, which constitutes a substantial part of CHD treatment, entails high costs that impose considerable financial burdens on patients and their families (3). This escalating healthcare expenditure presents a formidable challenge to national healthcare systems (4), prompting countries to seek methods for rationalizing healthcare costs (5), For instance, Germany’s German Diagnosis-Related Groups (G-DRG) System, the Accountable Care Organizations (ACOs) model in the United States, Japan’s Diagnosis Procedure Combination (DPC) System, and France’s Tarification à l’Activité (T2A) system, etc. These reforms have achieved some success in controlling healthcare costs and improving healthcare efficiency, yet they also give rise to certain problems and challenges, such as the transfer of high - cost cases and a decline in healthcare service quality (6–9). Australia also implemented the Activity-Based Funding (ABF) payment system nationwide during the 2011–2012 fiscal year. Research shows that ABF has achieved some success in controlling medical expenses. However, the implementation effects vary across states and hospitals. Some hospitals, in a bid to optimize fund allocation, have been selective in patient admission. Subsequently, Australia also carried out several Diagnosis-Related Groups (DRG) reforms to address the issues caused by the ongoing refinement of DRG (10, 11). The UK’s Payment by Results (PbR) system, which is based on DRG, also faces similar problems. Although it has contributed to improving hospital efficiency, it has also raised concerns about the potential impact on the quality of healthcare services (12).

In China, Basic Medical Insurance (BMI) coverage expanded to 1.3 billion people by 2021, prompting reforms in healthcare payment methods to address escalating healthcare costs (13). The traditional fee-for-service (FFS) model is inadequate, leading to service overutilization and heightened healthcare costs (14).

In 2019, the Chinese government initiated Diagnosis-Related Group (DRG) payment reforms to balance cost containment and healthcare efficiency. However, a one-year evaluation in pilot cities revealed that although the DRG reform reduced healthcare spending, it also increased patient hospitalization rates and neglected acutely ill patients (15–17). Thus, the Chinese government has been consistently exploring novel solutions, leading to the introduction of the Diagnosis-Intervention Packet (DIP) payment system, which categor patientsizes based on specific cases (18). The theoretical framework of the system originates from the “risk selection theory” of Germany’s DRG reform and the “institutional response hypothesis” of the ACO model in the US (19, 20). Compared with the “hospital - grading exemption mechanism” of Japan’s DPC system and the “global budget buffer design” of France’s T2A system, the particularity of China’s DIP reform lies in that it achieves a balance between total control of medical insurance funds and the autonomy of medical institutions through “dynamic adjustment of case - based scores” (21). China currently has a dual - track system of DRG and DIP payment methods. The DIP payment system is now being piloted in 71 cities (22). The DIP categorizes patients using ICD-10 and ICD-9-CM3 codes to form a disease portfolio, focusing on major disease diagnoses and treatment modalities (23).

The implementation of the DIP system in China demonstrates that DIP offers advantages over DRG in comprehensively covering healthcare services. DIP payments foster internal competition and stringent control among providers through the establishment of payment coefficients and “health insurance fund pools” (21). However, the effectiveness of DIP varies by region, depending on historical cost data to determine disease group weights, which leads to variations in implementation across regions.

The effectiveness of implementing DIP reforms varies across regions and types of healthcare organizations. Some studies have shown that DIP reforms significantly reduced average hospitalization and drug costs per patient in economically developed regions, but did not achieve the desired effect in other regions, and even led to an increase in patient healthcare costs in the short term (24, 25). Previous studies have examined the impact of payment reforms on other chronic conditions, but fewer have focused on coronary heart disease.

While emphasizing innovative payment methods, Chinese policymakers and researchers have also prioritized health equity. Within China’s health insurance system, UEBMI and URRBMI constitute the two primary components. Studies have demonstrated that disparities in population coverage, financing mechanisms, and reimbursement policies between these two health insurance systems can lead to health inequities among patients. Since DIP is a reform targeting healthcare providers, initial considerations did not include whether residents with different health insurance types are affected by health equity. Due to differences in contributions, benefit coverage, and medical expenditures between China’s UEBMI and URRBMI, budget caps were set based on health insurance types. At the time of DIP implementation, UEBMI had a sufficient budget, while URRBMI faced budget constraints. Consequently, whether innovative payment methods impact healthcare equity for patients with different health insurance types remains an important question for exploration (4).

In this study, a pilot city sample was selected from S city in central China, one of the initial pilot areas. UEBMI and URRBMI CHD patients in S city were selected to assess the impact of DIP policy implementation across various healthcare institutions on patients with both insurance types. This study aims to provide a deeper understanding of the practical application effects of the DIP payment method reform in different healthcare systems, offer a scientific basis for optimizing healthcare policies, enhance the efficiency of healthcare fund utilization, alleviate the economic burden on patients, and promote the rational allocation of healthcare resources.

## 2 Methods

### 2.1 Study design

City S, situated in Hubei Province in central China, was among the first cities in China to fully implement the DIP payment methodology across all healthcare organizations. Owing to the successful implementation of this pilot, City S has been designated as a model unit by the National Health Security Bureau. Given its pioneering and prominent status, this study utilizes data from the Medical Protection Bureau of City S to conduct the empirical analysis.

City S comprises 10 urban districts and 59 rural districts, with a resident population of 3.15 million and a 2023 GDP per capita of 73,489.10 RMB. These statistics indicate that the City S is at a medium level of economic and social development and is representative of a typical prefecture-level city in China. City S, as one of 71 pilot cities undergoing DIP reform, has a designated DIP disease catalog that includes 5,393 core diseases and 928 comprehensive diseases. Regarding health insurance, the city has 545,400 UEBMI participants and 2,459,100 URRBMI participants, with a social insurance coverage rate of 95.3%.

In January 2022, the local Medicare authority in City S formally implemented DIP payments for all providers in the city. To ensure the validity of this study, we conducted a comprehensive review of published literature and relevant policies to identify and consider any potential confounding policies that might affect hospital inpatient service behaviors. We selected interrupted time series (ITS) analysis as our research methodology. The main reason for using an interrupted time - series (ITS) design in this study is its unique policy evaluation advantages. First, the policy was city - wide and lacked a natural control group. Second, ITS models trends before and after intervention, distinguishing policy effects from time - related confounders like seasonal fluctuations and long - term trends. Lastly, ITS is widely used in payment reform evaluation and proven effective in health policy research (26–28). Based on the relevant policy documents, we used January 2022 as the intervention point for this study and employed an interrupted time series design to assess the impact of DIP payment reforms on hospital services for differently insured patients at all levels of hospitalization in City S. This provides insights into whether DIP reforms are likely to affect health equity between UEBMI and URRBMI hospitalized patients.

### 2.2 Data sources and samples

City S did not experience a major outbreak during the COVID-19 epidemic, thus its healthcare activities were minimally impacted, facilitating time series analysis. The data utilized in this study were sourced from the city’s Medicare information platform, which collated monthly Medicare claims data from January 2020 to December 2023 and anonymized the data to safeguard patient privacy. Each claims data record primarily included details such as the patient’s gender, type of health insurance, occupational status, length of hospitalization, discharge information, diagnosis, amount of Medicare reimbursement, and the hospital level in question. During the data cleaning phase, datasets with missing entries, irregular hospitalization or discharge lengths, and unusual expenditure figures were excluded. The final dataset used for our analysis comprised a total of 107,714 participants.

#### 2.2.1 Coronary heart disease

Coronary atherosclerosis (CHD) is a prevalent cardiovascular disease characterized by lipid deposition and plaque formation on the inner lining of coronary arteries, resulting in the narrowing or blockage of blood vessels. CHD is currently the leading cause of death globally.

Treatment for CHD primarily involves lifestyle modification, drug therapy, and, for patients with poor drug therapy outcomes, percutaneous coronary intervention or coronary artery bypass grafting. CHD has a substantial patient population and is less influenced by environmental factors such as seasons. Previous studies have conducted limited research on individual diseases. Therefore, this paper selects data coded as I25 in the ICD-10 disease codes and compiles it into a dataset following data cleansing.

#### 2.2.2 Types of health insurance

After the upgrade of China’s medical insurance system to an integrated level, the primary insurance types are UEBMI and URRBMI. UEBMI is medical insurance for urban employees, with premiums paid jointly by the organization and the individual. It primarily covers outpatient and hospitalization costs and is mandatory for all workers with regular employment. URRBMI, conversely, is medical insurance for urban and rural residents, encompassing both urban and rural populations. It is government-led, with voluntary individual participation, and primarily covers hospitalization costs and some outpatient expenses.

#### 2.2.3 Hospital level

In China, hospitals are categorized into three levels based on their functions, facilities, and technical capabilities: primary hospitals, secondary hospitals, and tertiary hospitals.

Primary hospitals are primary care hospitals or health clinics, mainly providing basic medical, preventive, rehabilitation, and health care services to communities. They are relatively small in scale, with fewer than 100 beds, and are primarily responsible for the initial diagnosis and treatment of common and frequently occurring diseases, as well as referring difficult and severe cases to higher-level hospitals.

Secondary hospitals are regional hospitals, usually county-level, district-level, or city-level hospitals, with bed numbers ranging from 101 to 500. They offer more comprehensive medical services, including specialized treatments and advanced surgeries. Secondary hospitals not only accept referrals from primary hospitals but also undertake certain teaching and research tasks.

Tertiary hospitals are large comprehensive hospitals, with more than 501 beds, providing high-level specialized medical services. Tertiary hospitals possess comprehensive capabilities in medical care, teaching, and research, are able to handle critical and difficult cases, and accept referrals from secondary hospitals.

### 2.3 Measurement variables

This study aims to examine the impact and differences of the DIP reform on healthcare expenditures, quality, and treatment for inpatients with two types of social health insurance across different hospital levels. To this end, the study employs three variables: hospitalization costs, hospital days, and out-of-pocket ratios, to conduct the necessary assessments. To account for the impact of inflation and other factors on hospitalization costs, we standardized hospitalization costs using China’s annual Consumer Price Index (CPI) with 2020 as the base year, ensuring that our findings reflect actual changes in healthcare costs. Given the typically skewed distribution of healthcare expenditure costs, we log-transformed the total healthcare costs per hospitalized patient. This transformation stabilizes the variance, facilitating statistical analysis and highlighting trends and outliers in healthcare spending more effectively. The number of hospitalization days was determined by calculating the variance between the patient’s admission and discharge dates. To ensure the accuracy of our statistical analyses, hospital days data following a normal distribution were appropriately processed. Out-of-pocket ratios were calculated on a cost-standardized basis, with total costs as the denominator and out-of-pocket costs as the numerator, to reflect the level of treatment for different health insurance types.

### 2.4 Statistical analysis

In this study, SPSS 24.0 software was used, with continuous variables presented as means ± standard deviations and categorical variables expressed as percentages. To analyze the impact of DIP policy implementation on patient characteristics and outcome variables in the sample, we performed t-tests and chi-square tests before and after the DIP reform, considering the effects of health insurance category and hospital level variables. Additionally, to assess the impact of the DIP policy intervention on patients’ total healthcare costs, hospital days, and out-of-pocket ratios, we employed a segmented regression model from interrupted time series analysis, with January 2021 designated as the intervention point. The general form of our ITS regression model is shown below:


$$Y_{t}=\beta_{0}+\beta_{1}\mathrm{Tt}+\beta_{2}\text{DIPt}+\beta_{3}\text{DIPtTt}+\varepsilon_{t}$$

In the interrupted time series analysis conducted in this study, the outcome variable Yt represents the outcome at a specific time point t. β0 estimates the baseline level of the outcome variable at the study’s inception. β1 represents the monthly slope of the outcome variable before the DIP policy intervention. β2 represents the immediate change in the outcome variable at the moment of the DIP reforms. Furthermore, β3 indicates the post-intervention change in the outcome variable compared to the expected trend change based on the pre-intervention period. The combination of β1 and β3 reflects the post-intervention trend. The time variable Tt spans from the study’s initiation to its conclusion, while DIPt is a binary variable indicating the occurrence of the DIP policy intervention (0 before the intervention and 1 after the intervention). Meanwhile, DIPtTt represents another time variable from the initiation of the DIP intervention to the study’s conclusion. Finally, εt represents the random error estimator at time t. To address potential autocorrelation in the model, we initially employed the Newey-West estimator with zero lags to fit an ordinary least squares (OLS) model. We subsequently tested for autocorrelation using the ‘actest’ command. If first-order autocorrelation is detected, the regression is re-estimated using the Prais-Winston method, and the validity of adjusting for autocorrelation is assessed using Durbin-Watson (DW) values. For second-order and higher-order autocorrelation, the Newey-West method was applied to adjust, specifying the necessary number of lags. For statistical significance tests, we used two - sided tests in the segmented regression model and set *α* = 0.05 as the significance threshold. All analyses were performed using STATA17.0.

## 3 Results

### 3.1 Sample characteristics

In this study, data pertaining to CHD patients with various insurance types in City S from 2020 to 2023 were selected. The data for employee health insurance were 9,955 before and 8,207 after the reform, and the data for URRBMI were 47,870 before and 41,682 after the reform. The table also presents the male-to-female ratio of the two insured populations, along with the number and percentage of visits to hospitals at all levels.

Supplementary Table 1 also presents the changes in medical costs, hospitalization days, and out-of-pocket ratio for insured UEBMI and URRBMI before and after the DIP reform, with significant differences observed in these variables before and after the reform. Specifically, the average hospitalization cost for patients enrolled in UEBMI decreased from 8.81 to 8.57, and for those enrolled in URRBMI, it decreased from 8.18 to 7.97. The average hospitalization time for patients enrolled in UEBMI decreased from 8.82 days to 7.78 days, and for those enrolled in URRBMI, it decreased from 8.24 days before the reform to 7.46 days after the reform. Regarding the out-of-pocket ratio, it decreased from 28.67 to 23.57% for patients enrolled in the UEBMI program, while it increased from 20.71 to 25.2% for those enrolled in the URRBMI.

### 3.2 Total cost of hospitalization

**Figure 1:**
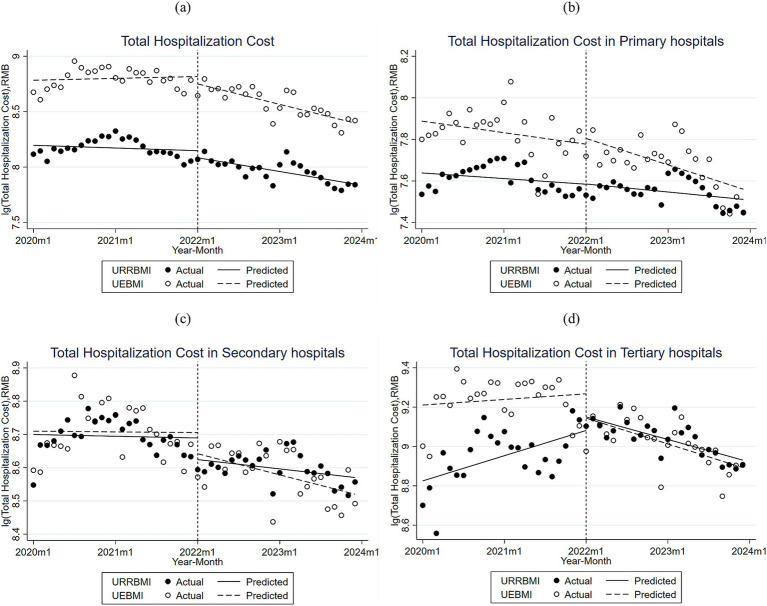
**(a)** Monthly trends in adjusted total cost per case for inpatients with UEBMI and URRBMI in all hospitals of City S; **(b)** Monthly trends in adjusted total cost per case for inpatients with UEBMI and URRBMI in primary hospitals of City S; **(c)** Monthly trends in adjusted total cost per case for inpatients with UEBMI and URRBMI in secondary hospitals of City S; **(d)** Monthly trends in adjusted total cost per case for inpatients with UEBMI and URRBMI in tertiary hospitals of City S.

The cost trend of healthcare expenditures for CHD patients enrolled in URRBMI and UEBMI in City S before and after the DIP reform is illustrated in Figure 1a and Supplementary Table 2. The initial levels of URRBMI and UEBMI were significantly different, and both experienced a decrease in overall costs after the reform. URRBMI hospitalization costs were initially at 8.196 before the DIP reform, with a non-significant decrease of 0.06 per month before the reform, and a significant decrease of 0.008 per month after the implementation of the DIP reform compared to the pre-intervention level. UEBMI hospitalization costs were initially at 8.783 before the DIP reform, with a non-significant decrease of 0.067 per year before the reform. After the implementation of the DIP reform, costs decreased significantly by 0.016 per month from the pre-intervention level.

Figures 1a–c and Supplementary Table 2 illustrate the cost trend of healthcare expenditures before and after the DIP reform for CHD patients enrolled in URRBMI and UEBMI in City S across hospitals of different levels. In primary hospitals, the initial level of URRBMI inpatient costs was 7.64, and the changes before and after the reform were not significant, but both were on a downward trend, with a greater decrease after the reform than before. In primary hospitals, the initial level of hospitalization costs for UEBMI was 7.89; the changes before and after the reform were not significant, but both were on a downward trend, with a greater decline after the reform than before.

In secondary hospitals, the initial level of hospitalization costs for patients enrolled in URRBMI was 8.699, which decreased significantly in the first month after the implementation of the DIP reform, and showed a more pronounced downward trend in subsequent years compared to the pre-implementation trend, although not significant. The initial level of hospitalization costs for patients using UEBMI was 8.709, with costs trending downward but not significantly both before and after the DIP reform, decreasing by 0.005 per month after the DIP reform compared to the pre-reform trend.

In tertiary hospitals, the initial level of hospitalization costs for URRBMI was 8.825, with a significant increase of 0.01 per month before the DIP reform, and a significant decrease of 0.02 per month after the implementation of the DIP reform compared to the pre-reform level. The initial level of hospitalization costs for UEBMI was 9.210, with a non-significant increase of 0.002 per month in hospitalization costs before the implementation of DIP, and a significant decrease of 0.013 per month in hospitalization costs after the implementation of the DIP compared to the pre-reform level. Costs decreased significantly by a margin of 0.013 per month from the pre-reform level.

### 3.3 Average length of hospitalization

**Figure 2:**
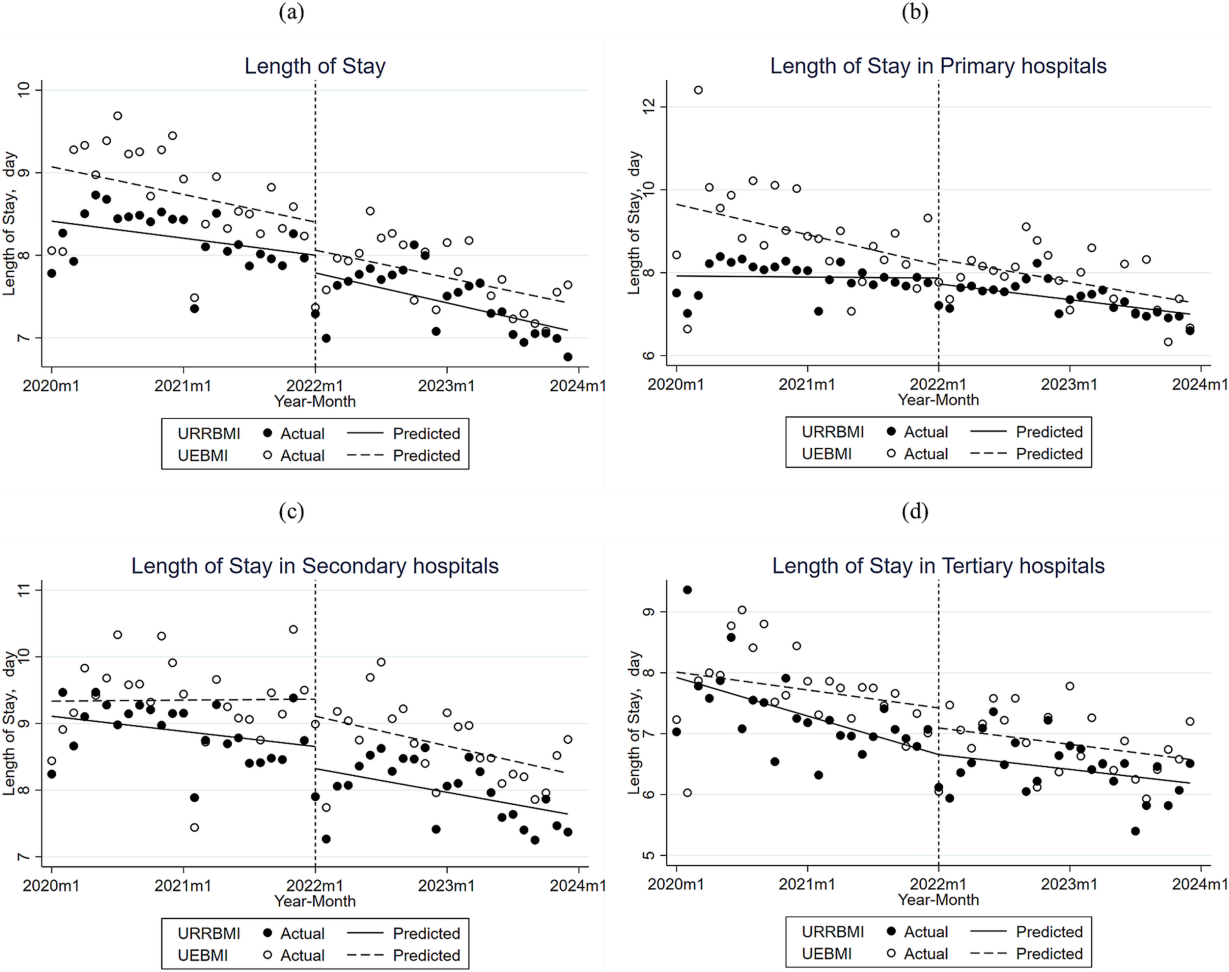
**(a)** Monthly trends in inpatient days for URRBMI and UEBMI inpatients in all hospitals of City S; **(b)** Monthly trends in inpatient days for URRBMI and UEBMI inpatients in primary hospitals of City S; **(c)** Monthly trends in inpatient days for URRBMI and UEBMI inpatients in secondary hospitals of City S; **(d)** Monthly trends in inpatient days for URRBMI and UEBMI inpatients in tertiary hospitals of City S.

The trend of the average length of hospitalization before and after the DIP reform for CHD patients enrolled in URRBMI and UEBMI in City S is illustrated in Figure 2a and Supplementary Table 3. The initial mean difference in hospitalization days between URRBMI and UEBMI was not significant, and the two were not significantly different before the DIP reform. However, in terms of trends, both experienced a decrease in overall length of stay after the DIP reform. The average number of total hospitalization days for URRBMI was 8.413 days before the DIP reform, with a non-significant decrease of 0.215 days per month in hospitalization days before the reform. After the implementation of the DIP reform, the number of hospitalization days did not change significantly from the pre-reform period, but showed an overall downward trend. UEBMI hospitalization days averaged 9.072 days before the DIP reform, with a non-significant monthly decrease of 0.341 days per month before the reform. After the implementation of the DIP reform, the number of hospitalization days did not change significantly from the pre-reform period, but showed an overall downward trend.

Figures 2b–d and Supplementary Table 3 illustrate the trend of hospitalization lengths for CHD patients enrolled in URRBMI and UEBMI in City S before and after the DIP reform across hospitals of different levels. The initial level of hospitalization days in primary hospitals using URRBMI was 7.922 days, and hospitalization days decreased before and after the implementation of the DIP reform, with a greater decrease after the implementation of the DIP reform. The initial level of inpatient days for primary hospitals using UEBMI was 9.651 days, showing a non-significant decrease by 0.06 days per month before the DIP reforms, and still showing a decreasing trend after the implementation of the DIP reforms, but at a slower rate than before the reforms.

The initial average length of hospitalization for URRBMI among cases attending secondary hospitals was 9.108 days, with a non-significant decrease in the length of hospitalization by 0.019 days per month before the implementation of the DIP reform, and an even greater decrease after the implementation of the DIP reform compared to the pre-reform period, with an additional decrease of 0.011 days per month. The initial average length of hospitalization for UEBMI was 9.336 days, with a slow non-significant upward trend in the length of hospitalization for UEBMI patients prior to the implementation of the DIP reform, and after the implementation of the reform, the length of hospitalization declined by a trend of 0.038 days per month less than prior to the implementation of the reform.

In tertiary hospitals, the initial average length of stay for URRBMI was 7.922 days, and before the DIP reform, the number of hospitalization days was decreasing significantly with a trend of about 0.05 days per month, and after the implementation of DIP, the number of hospitalization days, although decreasing, decreased more slowly than before the implementation of DIP. The initial average number of hospitalization days for UEBMI was 8.01 days, and before the DIP reform, the number of hospitalization days was decreasing with a trend of about 0.02 days per month, and after the implementation of the DIP, the number of hospitalization days, although decreasing, decreased with a slower trend compared to the pre-implementation period.

### 3.4 Out-of-pocket ratio

**Figure 3:**
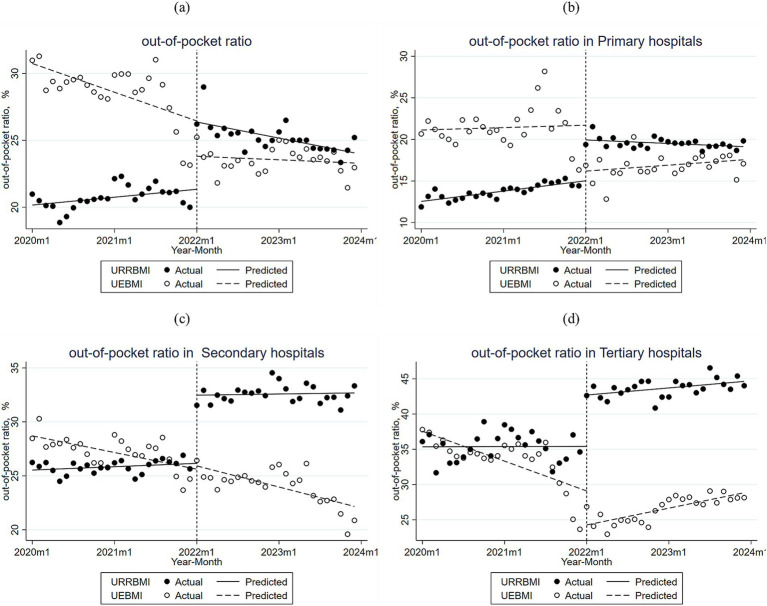
**(a)** Monthly trends in the proportion of out-of-pocket payments for inpatients with URRBMI and UEBMI in all hospitals of City S; **(b)** Monthly trends in the proportion of out-of-pocket payments for inpatients with URRBMI and UEBMI in primary hospitals of City S; **(c)** Monthly trends in the proportion of out-of-pocket payments for inpatients with URRBMI and UEBMI in secondary hospitals of City S; **(d)** Monthly trends in the proportion of out-of-pocket payments for inpatients with URRBMI and UEBMI in tertiary hospitals of City S.

As shown in Figure 3a and Supplementary Table 4, the trend of the average out-of-pocket ratio of CHD patients enrolled in URRBMI and UEBMI in City S before and after the DIP reform is demonstrated. The difference in the initial average between the URRBMI and UEBMI out-of-pocket ratios was significant, as was the difference in the pre-reform period. The difference remained significant in the first month of DIP reform implementation. The average out-of-pocket ratio for URRBMI was 20.16% before the DIP reform, and the change in the out-of-pocket ratio from month to month was not significant before the reform. In the year of implementation of the DIP reform, the out-of-pocket ratio increased by 5.02%, and then decreased by an average of 0.15% per year from the pre-reform trend. The average out-of-pocket ratio of UEBMI was 30.74% before the DIP reform, declining at a rate of 0.18% per month before the reform, and increasing slowly by 0.16% per month after the implementation of the DIP reform over the pre-reform out-of-pocket ratio.

Figures 3a–c and Supplementary Table 4 demonstrate the trend of out-of-pocket ratio for CHD patients enrolled in URRBMI and UEBMI in City S before and after the DIP reform across hospitals of different levels. In primary hospitals, the initial level of out-of-pocket ratio for URRBMI was 12.54%, and the out-of-pocket ratio showed a significant monthly increase in the pre-implementation period, with an increase of 4.92% in the month of implementation of the DIP reform, and the overall out-of-pocket ratio in subsequent months was higher than the pre-implementation period. Cases increased, but showed a certain downward trend. The out-of-pocket expense ratio for UEBMI in primary hospitals was initially 21.13%, decreasing by 5.53% immediately after the implementation of DIP, and the overall out-of-pocket expense ratio decreased in subsequent years compared to the pre-implementation level, but showed a certain upward trend.

In secondary hospitals, the initial level of out-of-pocket ratio for URRBMI was 25.54%, increasing significantly by 0.02% per month before the implementation of the reform, and increasing significantly by about 6.3% in the first month of the implementation of the DIP reform, with the general trend in the subsequent months of the implementation of the reform not significant compared to the pre-initial level, and the overall upward trend slowed down. The initial level of out-of-pocket expenses for UEBMI was 28.7%, decreasing significantly by 0.23% per month before the implementation of the reform, and the overall trend after the implementation of the DIP reform was the same as before the reform, with a greater decrease than before the implementation.

In tertiary hospitals, the initial level of the URRBMI out-of-pocket ratio was 35.36%, with a significant increase of about 7.3% in the first month of implementation of the DIP reform, and a non-significant monthly increase of 0.08% in the ongoing phase of implementation. The initial level of out-of-pocket ratio for UEBMI was 37.56%, showing a significant and gradual decrease in the pre-implementation level; in the first month of the implementation of the DIP reform, the out-of-pocket ratio decreased significantly by about 4.8%, and in the continuation phase of the program, the out-of-pocket ratio showed a slow increase, significantly different from the pre-implementation trend.

### 3.5 Robustness tests

Supplementary Tables 1–3 in the additional file show the results of the test using September 2021 as the intervention point to test the stability of the experiment. Among the results against the changes before and after the reform show that the results are insignificant, proving the reliability of the experiment and verifying the robustness of the experiment. Some of the results for the pre-intervention period show significant changes, which may be due to related policies such as the price adjustment of medical services.

## 4 Discussion

In this study, data pertaining to patients with CHD in City S from 2020 to 2023 were selected, encompassing two types of enrollment: employee health insurance (UEBMI) and resident health insurance (URRBMI). The data results indicate that hospitalization costs and hospitalization days in both UEBMI and URRBMI have declined compared with the pre-reform period, and the DIP reform has achieved significant results in controlling healthcare costs and hospitalization days. This finding echoes the “payment - method - insurance - type interaction effect” seen in France’s T2A reform. However, China’s DIP reform has led to a larger drop in costs, which may be related to hospitals adjusting their behavior due to the pressure of total control over medical insurance funds (29, 30). However, as in Australia’s ABF reform, DIP reform may also lead to hospitals selectively accepting patients, requiring further attention and study (11). However, in terms of out-of-pocket ratios, the performance of different health insurance types varies.

The DIP reform has demonstrated significant results in controlling treatment costs, consistent with the effects of previous DRG implementations (31, 32). The hospitalization costs of both URRBMI and UEBMI patients decreased after the reform, with a larger decrease in hospitalization costs for UEBMI, which may be related to both hospital choice and patient choice. In terms of the nature of the insurance itself, China’s URRBMI is primarily funded by the government and individuals, and is a universal bottom-up health insurance. Therefore, the amount of health insurance premiums paid is low, and the corresponding treatment is lower than that of those who pay the UEBMI (33). Hospital treatment costs are lower due to the low budget and large number of URRBMI patients. After the DIP reform, hospitals have to bear the excess due to the low cost of treatment and medication (34). The health insurance premiums for UEBMI are higher, so hospitals tend to choose more expensive treatments due to short-term income and other factors to make higher profits and obtain higher points in the next year’s point calculation (4). This choice will, to a certain extent, cause inequality in health among populations (35). Secondly, on the subjective side, the UEBMI population may also have higher budgets for expected treatment costs due to better treatment, resulting in treatment costs being at a higher level (36, 37). Regarding the difference between the cost of residential health insurance and that of UEBMI, some studies have shown that although the integration of residential health insurance reforms has promoted health equity, the impact on different regions and income groups varies, and the low-income group still suffers from more serious health inequalities (38). This phenomenon resembles the “high - cost case transfer” in Germany’s DRG reform and the divergent evolution of commercial and national health insurance under Japan’s DPC system (39). It is recommended to refer to the risk - adjustment mechanism in the US ACO model and apply weight correction based on the Charlson Comorbidity Index for URRBMI patients admitted to hospitals (40).Previous studies on DRG have also mentioned the existence of reforms that have reduced drug costs but increased overall treatment costs for chronic conditions. Since the research on DIP in this study is biased toward its short-term effects, it is possible to conduct a long-term follow-up study to examine the total cost of treatment for chronic conditions over multiple years (41). The downward trend in hospitalization costs is reflected in different levels of hospitals. These results suggest that the DIP reform has been effective in curbing the increase in hospitalization costs in different levels of hospitals, especially in primary and secondary hospitals. Long - term studies on Japan’s DPC and Germany’s DRG reform show a 3 to 5-year lag between payment reform and healthcare - quality changes, indicating that our three year study may be limited by an “effect-observation window period” (7, 39).The fact that primary and secondary hospitals have better cost containment results than tertiary hospitals can be attributed to several factors. Previous studies have shown that DRG has different cost-control effects on different levels of hospitals (42, 43), Primary and secondary hospitals are more advantageous in terms of cost control due to their simple cost structure, single type of disease treated, and more standardized treatment protocols. Compared to DRG, hospitals have more initiative in payment coefficients during the DIP reform, and primary and secondary hospitals can adjust payment coefficients by optimizing historical data to reduce inpatient costs. Tertiary hospitals, on the other hand, are less effective in controlling costs due to the complexity of patients’ illnesses, the volume of historical data, and the complicated cost structure. However, studies have also shown that tertiary hospitals are able to avoid sacrificing profitability by lowering the price of services to attract patients in the competition due to their better cost control ability (44).

The results on length of stay before and after the DIP reform also provide some evidence for the corresponding view on hospitalization costs described above. The average length of stay for both residents’ and UEBMI declined after the reform, and the declining trend was reflected in different levels of hospitals. Specifically, the decline in hospital days was more pronounced for UEBMI patients than for URRBMI patients. This result is somewhat inconsistent with previous studies (4, 45, 46), which may be related to factors such as the study’s regional and demographic and health resource allocations (47), but both reflect good cost control in DIP. The reason for the larger decrease in hospital days for UEBMI patients than for URRBMI patients after the implementation of the DIP payment method may be related to several factors. First, the post-payment price of UEBMI is usually higher than that of URRBMI, which incentivizes hospitals to be more motivated to improve efficiency by reducing the number of inpatient days in treating UEBMI patients, thus reducing the use of healthcare resources without compromising the quality of treatment. Second, from the patient’s perspective, employee patients are mostly active workers, relatively young, and usually have milder conditions, so it is easier to achieve a reduction in the number of hospitalization days under the DIP payment method. URRBMI patients, on the other hand, include the unemployed, farmers, and the older adult. These groups may have more complex conditions and require longer hospitalization. In addition, hospitals may prefer UEBMI patients in resource allocation due to the higher post-payment price of UEBMI, which may result in less significant reductions in hospital days for URRBMI patients than for employee patients (4), These factors together resulted in a greater decrease in hospital days for UEBMI patients after the implementation of the DIP payment method. The data on length of stay in different levels of hospitals showed a high degree of consistency with the reduction in hospitalization costs. From previous studies related to DRG, it was found that hospitals may increase the number of hospitalizations to obtain additional subsidies (16, 48). In this study, because readmission rates were not included in the study variables, it was not possible to ensure whether any hospitals obtained corresponding benefits by disaggregating hospitalizations.

The DIP reform showed different effects in terms of out-of-pocket expenses. The out-of-pocket ratio for URRBMI increased after the reform, while the out-of-pocket ratio for UEBMI decreased after the reform. Specifically, the out-of-pocket ratio for URRBMI was 20.16% before the DIP reform, rose by 5.02% after the reform, and subsequently declined by an average of 0.15% per year from the pre-reform trend. The out-of-pocket ratio for UEBMI was 30.74% before the DIP reform, and has been rising slowly by 0.16% per month since the reform compared to the pre-reform out-of-pocket ratio. The increase in the out-of-pocket ratio of the URRBMI may be related to the corresponding policy of the health insurance pool. Previous studies have pointed out that a single health care provider can sometimes lead to an increase in out-of-pocket medical expenses or an increase in other medical expenditures (49, 50). The lack of a comprehensive and rational policy mechanism may also lead hospitals to cut reimbursement budgets to cope with the risk (51). Because the aging of Chinese society has led to the expansion of the URRBMI group, in order to ensure the sustainability of the health insurance fund, some regions have made certain policy adjustments, such as raising the starting line and lowering the reimbursement rate to cope with the risk of bottoming out the fund (52), and these adjustments may directly lead to an increase in the out-of-pocket expenses of the URRBMI. However, when linked to the overall cost trend of URRBMI, the out-of-pocket costs have still decreased compared to the pre-reform period. The increase in out-of-pocket expenses may also be related to the characteristics of the population enrolled in the URRBMI, which has a higher probability of suffering from other chronic and basic diseases due to its older age on average, while the persistence of medication for basic diseases and the adjustments of some drug catalogs may also lead to a certain degree of increase in the out-of-pocket expenses. Among different levels of hospitals, the changes in the out-of-pocket ratios of URRBMI patients and UEBMI patients in level 1 hospitals and level 2 hospitals are basically the same as the overall. The out-of-pocket ratios for URRBMI in tertiary hospitals, on the other hand, showed a trend of increasing in the month after the reform and then decreasing on a monthly basis. Previous studies have shown that the risk adjustment mechanism established will be more compatible with the payment standards of tertiary hospitals, thus providing more reasonable treatment and services to patients while alleviating the pressure of cost control (53), a finding consistent with the results of this study. The decrease in out-of-pocket ratios for URRBMI patients in tertiary hospitals after the implementation of DIP is also attributed to the fact that tertiary hospitals usually have a higher institutional factor in the DIP reform and are able to receive more financial support for Medicare payments. The health insurance department has also given certain policy support and fund tilts to tertiary hospitals to encourage them to admit and treat critically ill patients and difficult cases, and this support has enabled tertiary hospitals to obtain more funds in health insurance payments, thus reducing the out-of-pocket proportion of patients.

This study has its strengths. First, the data covered all hospitalization claims from January 2020 to December 2023 for all patients with coronary artery disease in the city. This ensured the adequacy of the assessment data. Second, all data were obtained from the hospitalization claims records of the S. City Health Protection Bureau, which ensured the quality of the data. Third, ITSA of the corresponding outcome variables for UEBMI and URRBMI hospitalized patients allowed for a more effective investigation of the respective impacts before and after the DIP reform. This study also has its limitations. First, it was obtained from only one city, a single source. Second, it lacks its own control group to make its own comparison. Third, because the variables are more crude, it may ignore the influence of other factors and cannot find the influence mechanism of more potential factors.

## 5 Conclusion

The DIP reform has achieved significant results in controlling hospitalization costs and hospital days, especially in Primary and secondary hospitals. The decline in hospitalization costs and hospital days reflects the short-term and more favorable cost-control effects of the DIP reform. The DIP reform has performed differently in terms of out-of-pocket ratios. Out-of-pocket ratios for URRBMI increased after the reform, while those for UEBMI decreased. Among the different levels of hospitals, Primary and secondary hospitals experienced larger declines in hospitalization costs and length of stay, which were attributed to their simple cost structures, standardized treatment protocols, and cost-control incentives under the DIP payment methodology. The downward trend in the length of stay in Tertiary hospitals slowed down after the reform, but the out-of-pocket ratio of URRBMI patients instead declined after the reform, which was mainly attributed to the fact that Tertiary hospitals had higher institutional coefficients under the DIP reform and were able to obtain more financial support for health insurance payments, while the policy support and fund tilting of health insurance departments toward Tertiary hospitals also lowered the out-of-pocket ratio of patients.

Overall, the DIP reform has effectively reduced the financial burden for patients and enhanced the operational efficiency of hospitals. When further promoting the DIP reform, policymakers should consider the fairness of medical treatment for patients with different types of health insurance and other circumstances, and seek the optimal solution whereby patients with different types of health insurance can receive good medical services, so as to optimize the provision of medical services while ensuring the universality and fairness of medical treatment. Future research can combine clinical - quality metrics, like 30 - day readmission rates, to build a DIP composite - performance index. It can also apply mixed - methods to explore how payers and patients adapt to payment rules.

## Data Availability

The datasets presented in this article are not readily available because it is from a public institution, institutional review is required. Requests to access the datasets should be directed to Yingying Tao, 15168340102@163.com.
