# Supplementary material for: A study of the impact of DIP payment reform on coronary heart disease hospitalization costs and equity
Source: Front Public Health. 2025 Jun 9;13:1567838. doi: 10.3389/fpubh.2025.1567838 (PMC12183219; doi:10.3389/fpubh.2025.1567838)
Supplement: Supplementary file 1 [file Table_1.docx]

Supplementary Material

# Supplementary Tables

Table 1 Robustness Tests of ITSA for Hospitalization Cost Variables

|  | Lg(Total expenditurepercase) | Baselinemonthlyslope(β_1_) | Stepchange(β_2_) | Monthlyslopechange(β_3_) | Constant(β_0_) |
| --- | --- | --- | --- | --- | --- |
|  |  | Estimate(95%CI) | Estimate(95%CI) | Estimate(95%CI) | Estimate(95%CI) |
| URRBMI | All hospitals | 0.002(-0.003,0.007) | -0.121(-0.196,-0.045) | -0.011(-0.017,-0.006) | 8.165(8.115,8.215)*** |
|  | Hospital level |  |  |  |  |
|  | Tertiary hospitals | 0.008(-0.001,0.018) | 0.135(-0.022,0.248) | -0.016(-0.026,-0.006) | 8.842(8.718,8.966)*** |
|  | Secondary hospitals | 0.002(-0.003,0.006) | -0.081(-0.131,-0.031) | -0.004(-0.009,0.000) | 8.685(8.629,8.740)*** |
|  | Primary hospitals | -0.001(-0.004,0.004) | -0.049(-0.113,0.149) | -0.002(-0.007,0.003) | 7.623(7.575,7.671)*** |
| UEBMI | All hospitals | 0.006(-0.001,0.012) | -0.106(-0.186,-0.025) | -0.019(-0.026,-0.012) | 8.752(8.673,8.831)*** |
|  | Hospital level |  |  |  |  |
|  | Tertiary hospitals | 0.009(0.001,0.017) | -0.192(-0.281,-0.103) | -0.019(-0.028,-0.010) | 9.164(9.052,9.276)*** |
|  | Secondary hospitals | 0.003(-0.003,0.008) | -0.090 (-0.169,-0.011) | -0.007(-0.014,-0.001) | 8.690(8.619,8.761)*** |
|  | Primary hospitals | -0.004(-0.013,0.003) | 0.034(-0.101,0.171) | -0.005(-0.014,0.004) | 7.891(7.828,7.953) |

Table 2 Robustness Tests of ITSA for Hospitalization Length Variables

|  | **Length of stay** | **Baselinemonthlyslope(β_1_)** | **Stepchange(β_2_)** | **Monthlyslopechange(β_3_)** | **Constant(β_0_)** |
| --- | --- | --- | --- | --- | --- |
|  |  | **Estimate(95%CI)** | **Estimate(95%CI)** | **Estimate(95%CI)** | **Estimate(95%CI)** |
| URRBMI | Allhospitals | -0.017(-0.041,0.006) | -0.101(-0.468,0.266) | -0.016(-0.045,0.011) | 8.415(8.102,8.727)*** |
|  | Hospital level |  |  |  |  |
|  | Tertiary hospitals | -0.061(-0.110,-0.012) | 0.105(-0.437,0.647) | 0.036(-0.017,0.088) | 7.979(7.314,8.643)*** |
|  | Secondary hospitals | -0.027(-0.059,0.004) | 0.004(-0.518,0.526) | -0.013(-0.052,0.026) | 9.167(8.757,9.577)*** |
|  | Primary hospitals | 0.002(-0.027,0.032) | -0.123(-0.477,0.231) | -0.033(-0.065,0.001) | 7.889(7.479,8301)*** |
| UEBMI | All hospitals | -0.024(-0.066,0.017) | -0.286(-0.789,0.218) | -0.009(-0.054,0.035) | 9.046(8.489,9.603)*** |
|  | Hospital level |  |  |  |  |
|  | Tertiary hospitals | -0.005(-0.057,0.048) | -0.651(-1.187,-0.115) | -0.015(-0.072,0.042) | 7.875(7.115,8.639)*** |
|  | Secondary hospitals | -0.014(-0.054,0.025) | 0.271(-0.431,0.972) | -0.034(-0.082,0.015) | 9.444(8.943,9.946)*** |
|  | Primary hospitals | -0.066(-0.173,0.039) | 0.140(-0.869,1.148) | 0.023(-0.088,0.134) | 9.692(8.231,11.154)*** |

**P*<0.05; ***P*<0.01; ****P*<0.001.

Table 3 Robustness Tests of ITSA for Out-of-Pocket Ratio Variables

|  | **Out-of pockets expenses,**(%) | **Baselinemonthlyslope(β_1_)** | **Stepchange(β_2_)** | **Monthlyslopechange(β_3_)** | **Constant(β_0_)** |
| --- | --- | --- | --- | --- | --- |
|  |  | **Estimate(95%CI)** | **Estimate(95%CI)** | **Estimate(95%CI)** | **Estimate(95%CI)** |
| URRBMI | All hospitals | 0.09(0.04,0.13) | 2.57(0.34,4.81) | -0.05(-0.18,0.07) | 19.90(19.28,20.53)*** |
|  | Hospital level |  |  |  |  |
|  | Tertiary hospitals | 0.02(-0.14,0.17) | 3.88(0.56,7.20) | 0.23(0.01,0.45) | 35.27(33.47,37.05)*** |
|  | Secondary hospitals | 0.02(-0.02,0.06) | 3.97(1.88,6.07) | 0.13(-0.01,0.25) | 25.57(25.07,26.08)*** |
|  | Primary hospitals | 0.10(0.07,0.15)*** | 3.17(1.31,5.03) | -0.02(-0.14,0.08) | 12.49(11.94,13.05)*** |
| UEBMI | All hospitals | -0.04(-0.12,0.04) | -4.88(-6.20,-3.56) | 0.01(-0.07,0.11) | 29.81(28.95,30.66)*** |
|  | Hospitallevel |  |  |  |  |
|  | Tertiary hospitals | -0.16(-0.27,-0.04)** | -8.33(-10.51,-6.16) | 0.31(0.17,0.45) | 36.21(35.11,37.31)*** |
|  | Secondary hospitals | -0.06(-0.13,0.01) | 1.25(-2.41,-0.08) | -0.06(-0.15,0.04) | 27.41(27.93,29.07)*** |
|  | Primary hospitals | 0.17(0.02,0.32) * | -6.66(-9.52,-3.70) | -0.17(-0.35,0.01) | 20.07(18.92,21.23)*** |

**P*<0.05; ***P*<0.01; ****P*<0.001.
